# Safety-first TCR-T: AI-guided specificity, base-editing to prevent mispairing, and prioritizing public neoantigens

**DOI:** 10.3389/fimmu.2026.1754735

**Published:** 2026-01-23

**Authors:** Yue Zhang, Yan Liu, Qian Wang, Yuanyuan Li, Xiaolin Kong

**Affiliations:** Hematology Department, Zibo Central Hospital, Zibo, China

**Keywords:** AI-guided specificity prediction, base editing, public neoantigens, TCR mispairing prevention, TCR-T cell therapy

## 1 Introduction

T cell receptor-engineered T cell (TCR-T) therapy has emerged as one of the most promising frontiers in adoptive cell therapy, offering the ability to target intracellular antigens presented by major histocompatibility complex (MHC) molecules and thereby expanding therapeutic reach beyond the limits of chimeric antigen receptor (CAR)-T cells (1, 2). The clinical success of CAR-T therapy in hematologic malignancies further underscores the need for TCR-based strategies to overcome the unique barriers presented by solid tumors (3, 4). Over the past decade, encouraging clinical data, such as responses against melanoma, synovial sarcoma, and multiple epithelial cancers, have highlighted the transformative potential of this strategy (5).

Clinically, early TCR-T trials against melanoma, synovial sarcoma, and multiple epithelial cancers have produced durable responses in a subset of patients, and a MAGE-A4–directed product (afamitresgene autoleucel) has recently achieved regulatory recognition, signaling that TCR-based therapies are entering a more mature translational phase (6). At the same time, development has been punctuated by high-profile toxicity events, such as titin cross-reactivity of affinity-enhanced MAGE-A3–directed T cells and other off-tumor/on-target injuries, which have left a lasting imprint on the perception of risk in this modality (7). Yet, despite these advances, both therapeutic efficacy and safety remain central barriers to the broad implementation of TCR-T therapy, with challenges in persistence, target selection, and pharmacokinetics evolving in parallel with concerns regarding off-target toxicity. Unlike CAR-T cells, TCR-T therapies carry unique risks, including TCR mispairing with endogenous chains, cross-reactivity with structurally similar peptides on normal tissues, and unpredictable off-target toxicities that may not be fully anticipated in preclinical models (7, 8). These challenges underscore an urgent need for a systematic framework that prioritizes safety without sacrificing therapeutic efficacy. Importantly, our intention is not to suggest that safety represents the final outstanding hurdle in TCR-T development; rather, we emphasize that safety-by-design must advance alongside ongoing efforts to improve efficacy, durability, and pharmacologic performance of TCR-based agents. However, current safety-evaluation strategies remain fragmented, experimental assays capture only a narrow slice of the peptide landscape, gene-editing approaches introduce new genomic risks, and antigen-selection pipelines often lack scalability, contributing to a persistent gap between TCR-T’s therapeutic promise and its real-world performance — a gap that reflects parallel limitations in both efficacy optimization and safety assurance. Notably, cross-reactivity liabilities are not unique to cellular TCR-T products: TCR-mimic (TCRm) or TCR-like antibodies that bind peptide-HLA (pHLA) complexes—including neoantigen-HLA targets—are emerging as “off-the-shelf” biologics (such as T cell engagers or ADCs), and they face the same fundamental risk of off-target pHLA recognition as bona fide TCRs (9–11).

In this Opinion, we argue that the next generation of TCR-T therapy must adopt a “safety-first” paradigm, structured around three complementary pillars. First, the integration of artificial intelligence (AI)-driven specificity assessment can serve as an initial safeguard, filtering out receptors with high cross-reactivity potential and guiding rational selection before costly and risky clinical deployment (12, 13). Second, precise base-editing approaches provide a robust means of preventing TCR mispairing by removing endogenous receptor chains while avoiding the collateral damage associated with traditional nuclease-based editing (14). Third, the strategic prioritization of public neoantigens (shared, recurrent mutations or aberrant gene products across diverse patient populations) offers a path to scalable, standardized, and regulatorily favorable therapies (15, 16). Importantly, these three components address distinct failure points in the current TCR-T pipeline: AI mitigates hidden cross-reactivity, base editing reduces receptor mispairing, and public neoantigen selection improves manufacturability, which highlighting their interdependence rather than functioning as isolated innovations.

**Figure 1:**
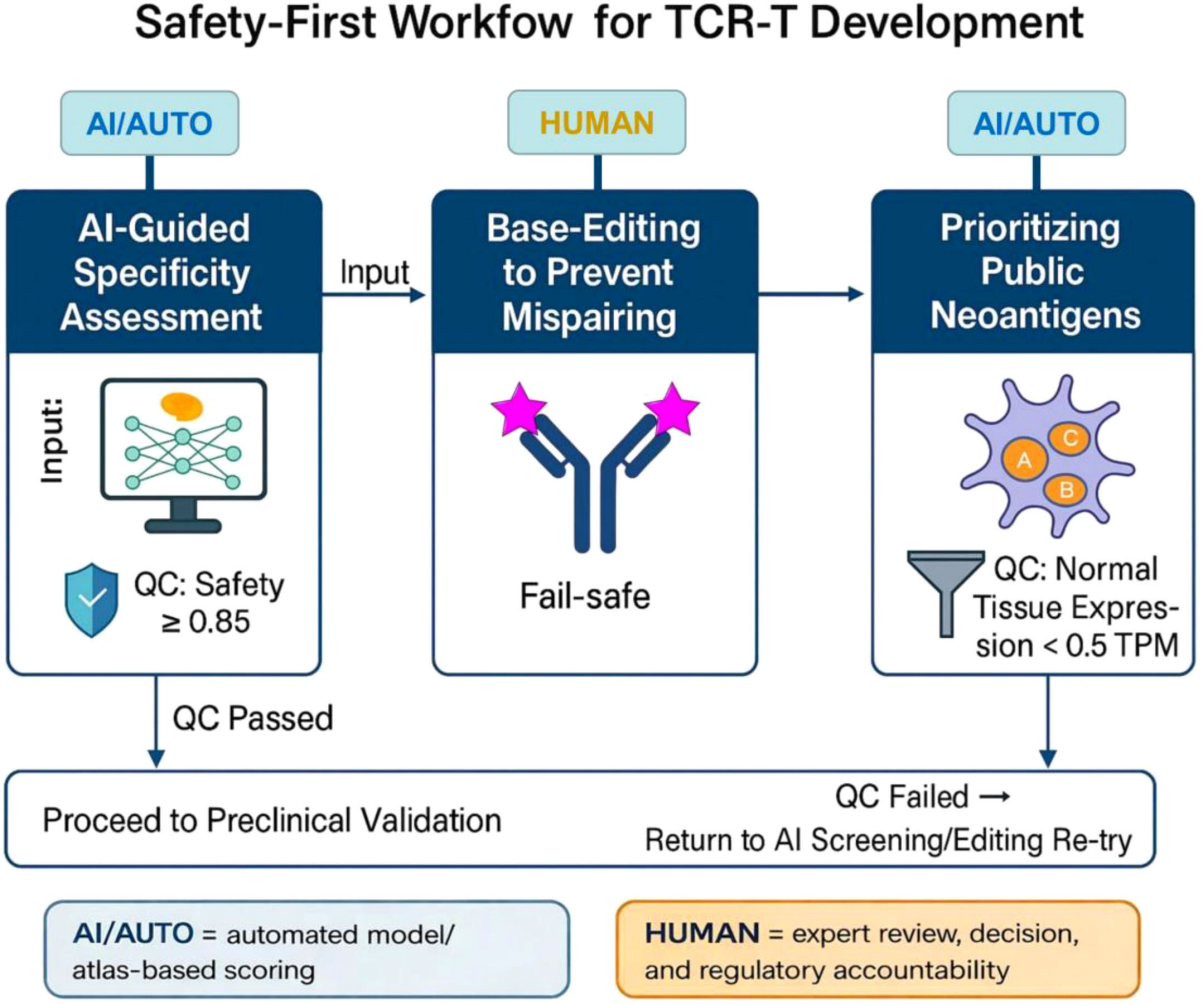
Safety-first workflow for TCR-T development. A schematic representation of a safety-oriented engineering pipeline for TCR-T cell therapy. The process integrates three major modules: (1) *AI-guided* sp*ecificity assessment* to identify cross-reactivity risks and ensure antigen recognition safety (QC: Safety ≥ 0.85, a conservative illustrative high-confidence gate to be probability-calibrated and anchored to outcome-linked cross-reactivity/toxicity cases, with uncertainty-aware filtering for out-of-distribution epitopes); (2) *Base-editing to prevent mispairing* of endogenous and exogenous TCR chains, equipped with a fail-safe mechanism to terminate unsafe edits; and (3) *Prioritizing public neoantigens* with minimal expression in normal tissues (QC: < 0.5 TPM, a tier-1 triage filter consistent with common atlas “below-cutoff/absent” conventions). Quality control (QC) gates are established at each step. Passing QC allows progression to preclinical validation, while failed QC returns candidates to re-screening or editing re-try. Transcripts per million (TPM) represents a normalized RNA-expression metric used to compare gene expression across tissues when assessing off-tumor antigen risk.

The diagram in **Figure 1** summarizes this three-stage framework, in which AI-guided specificity screening, precise base-editing, and the prioritization of public neoantigens are integrated into an iterative safety pipeline with explicit quality-control gates. Qualified receptors progress through these gates toward preclinical validation, while unsafe candidates are recycled for re-design. Such a structured, safety-first workflow is largely absent from current TCR-T development, and our Opinion aims to stimulate discussion on how adopting this type of pipeline could help TCR-T therapies transition from high-risk experimental interventions to standardized, widely accessible treatments. Rather than a neutral literature update, this article presents an author-driven position advocating a three-stage ‘safety-first’ pathway for next-generation TCR-T development. **Figure 1** also distinguishes AI-automated scoring/triage steps from human-in-the-loop decision points to emphasize auditability and regulatory accountability.

## 2 AI-guided specificity assessment

Beyond engineered TCRs, antibody-based TCR mimics (TCRm; also termed TCR-like antibodies) have been developed to recognize specific peptide-HLA complexes, including cancer neoantigen-HLA targets derived from recurrent mutations. These modalities can be deployed as bispecific T cell engagers, ADCs, or as binding domains in CAR-like formats, but they remain vulnerable to unintended binding to structurally related off-target peptides presented by HLA. Consistent with this, computational and structural analyses suggest that some TCRm antibodies may exhibit reduced peptide selectivity compared with native TCRs, underscoring the need for proteome-wide off-target triage and high-throughput peptide scanning during lead selection and safety testing (10, 11). The specificity of TCR recognition is both its greatest strength and its most formidable liability (17). While high-affinity TCRs can detect peptide–MHC complexes at exceedingly low densities, subtle structural similarities between tumor-associated and normal self-peptides may result in catastrophic off-target effects (18). Indeed, several widely cited toxicity events in early clinical programs have underscored the need for more systematic approaches to evaluating cross-reactivity risk. In particular, the cardiotoxicity associated with a MAGE-A3–directed TCR that cross-reacted with titin arose from an artificially affinity-matured receptor rather than a native TCR clone (19), illustrating how supraphysiologic affinity engineering may amplify the probability of unintended peptide recognition. We acknowledge that such extreme off-target recognition is less likely for neoantigen-specific TCRs derived from endogenous T-cell repertoires, as these cells have already passed thymic negative selection and therefore exhibit a lower baseline propensity to cross-react with structurally related self-antigens such as titin (20). At the same time, however, even TCRs originating from native repertoires may encounter low-density self-peptide presentation or cryptic peptide analogs that are insufficiently revealed by conventional screening assays, which motivates the development of more comprehensive and quantitative strategies to estimate proteome-wide specificity risk prior to clinical deployment (21). In this Opinion, we contend that AI-guided specificity assessment should not merely complement existing experimental screening, but should operate as the first safety gate in TCR-T development, providing a systematic and quantitative filter for cross-reactivity risk across the proteome. Current experimental pipelines, including alanine-scanning mutagenesis and peptide libraries, are resource-intensive and fail to capture the full spectrum of peptide diversity across the human proteome (22). This gap necessitates new computational solutions capable of predicting potential cross-reactivity before clinical translation. From a safety perspective, the central unmet need is not merely to identify additional candidate targets, but to generate a calibrated, proteome-wide estimate of off-target risk that can be used as an explicit decision gate in TCR development.

Models such as DeepTCR, TITAN, ImRex, and hybrid attention-based architectures leverage large-scale TCR–pMHC datasets to learn recognition patterns that are not easily accessible through traditional bioinformatics (23, 24). Importantly, these models can be trained on both structural and sequence-based data, allowing them to generalize across allelic variants and diverse peptide repertoires. Early results suggest that AI-based predictors can flag receptors with high probabilities of cross-reactivity, enabling researchers to deselect unsafe candidates prior to wet-lab validation. In practice, these tools can be used to assign each candidate receptor a quantitative “specificity risk score” based on predicted binding to off-target peptide–MHC complexes, enabling tiered triage (for example, deprioritizing receptors within the highest-risk decile for experimental follow-up). Rather than replacing functional assays, such scores can rationally constrain the search space, ensuring that limited experimental capacity is focused on clones most likely to meet predefined safety thresholds.

While most existing TCR–pMHC prediction models were originally developed in the context of epitope discovery and receptor selection, their outputs can be repurposed in a conceptually distinct way when used within a safety-first engineering pipeline. In discovery-oriented applications, predicted binding probabilities are used to prioritize candidate epitopes or TCRs for further development (23, 25). In contrast, in the safety-screening use case that we emphasize here, the same predictive scores are operationalized as a filtering mechanism: models are applied across large peptide libraries to identify receptors that exhibit a higher likelihood of recognizing off-target self-peptides, thereby enabling early de-prioritization of high-risk clones. Importantly, we view this as a complementary, design-stage triage layer rather than a substitute for downstream functional or structural validation, but one that can systematically reduce the probability that unsafe receptors progress into costly preclinical or clinical evaluation.

Conceptually, most current TCR–pMHC prediction models are trained on paired receptor–ligand datasets generated from multimer-based sorting, high-throughput display platforms, or curated repositories that aggregate validated TCR–epitope interactions (26). In these settings, the inputs typically include CDR3 sequences (and often V/J gene usage) for the TCR, the peptide sequence, and an HLA “pseudo-sequence” or allele identifier, with some models additionally incorporating structural encodings of the peptide–MHC groove. The outputs are continuous or categorical scores reflecting the likelihood or strength of binding for a given TCR–peptide–HLA combination (12). To estimate off-target risk, a candidate therapeutic TCR can be scored in silico against large peptide libraries derived from the human proteome or tissue-restricted subsets, and summary metrics—such as the number of predicted off-targets above a given affinity threshold, the highest-risk off-target hit, or the aggregate probability mass of non-intended binders—can be used to define an explicit “specificity risk score” that feeds into the safety gate of the engineering pipeline.

Nevertheless, it is critical to acknowledge that AI cannot yet function as a stand-alone arbiter of safety. Datasets remain biased toward well-studied epitopes, limiting generalizability across rare HLA alleles or poorly characterized tumor antigens (27). Concretely addressing the unseen-epitope problem requires both methodological and community-level changes. First, models should be evaluated under epitope-/HLA-disjoint splits and report out-of-distribution (OOD) performance, as recent benchmarking studies highlight strong data dependency and limited robustness (26). Second, safety screening should be uncertainty-aware: predictions in low-confidence or OOD regimes should trigger an explicit “abstain/flag” decision rather than being treated as safe. Third, the field can implement active-learning loops, where high-uncertainty peptide/HLA neighborhoods are prioritized for high-throughput experimental scanning to generate informative positives and “hard negatives,” which are then fed back to recalibrate models. Finally, coordinated public benchmarks with standardized negative generation and external validation (IMMREP initiatives) can reduce dataset bias and make threshold calibration more transferable across cohorts (28).

As a result, models that perform well on benchmark datasets can show substantial drops in accuracy when evaluated on independent cohorts or truly unseen epitope–TCR combinations. In addition, many current predictors are poorly calibrated—their output scores do not map linearly onto real-world probabilities of cross-reactivity—making it difficult to define clinically meaningful risk thresholds. Here, we use “Safety Score > 0.85” as a conservative, operational gate rather than a universal clinical cutoff. The value should be set only after probability calibration on held-out data and stress-tested against known ‘near-miss’ liabilities. For example, engineered-TCR programs that produced fatal off-tumor toxicities from unexpected peptide mimicry (7, 29). Retrospectively, the intent is that constructs linked to severe clinical signals (such as cardiotoxicity or neurotoxicity) cluster below this gate, whereas candidates with clean normal-tissue panels remain above it. In practice, 0.85 can be chosen as a high-confidence point on ROC/PR curves that prioritizes minimizing false negatives (missed cross-reactivity), then paired with orthogonal wet-lab assays and periodic recalibration as new epitopes accumulate (30).

By positioning AI as the first gatekeeper in the TCR engineering pipeline, we can reduce the probability of advancing high-risk clones into clinical development, streamline preclinical workflows, and create a culture of safety-by-design. This shift not only minimizes patient risk but also accelerates the translational path by improving regulatory confidence in candidate receptors.

## 3 Base-editing to prevent TCR mispairing

One of the most persistent safety challenges in TCR-T therapy is the risk of mispairing between introduced transgenic TCR chains and endogenous TCR α/β chains. Such mismatches can generate hybrid receptors with unpredictable specificities, potentially triggering autoreactivity or diluting the therapeutic potency of engineered cells (31). In several clinical programs, relatively simple engineering approaches — including codon optimization and the use of murine or otherwise modified constant regions to promote preferential pairing between transduced TCR chains and to disfavor endogenous pairing — have already proven effective in substantially reducing mispairing, while maintaining product feasibility and manufacturing simplicity (32). As the field matures, it has become evident that the risk of TCR chain mispairing can be mitigated through multiple engineering strategies rather than a single mandatory intervention.

Gene editing technologies have opened new opportunities to tackle this problem at its root. CRISPR/Cas9- or TALEN-mediated disruption of endogenous TCR genes has shown promise in producing “monospecific” T cells, but nuclease-based methods carry inherent risks of double-strand breaks, chromosomal translocations, and p53 activation (33, 34). Recent studies have also shown that nuclease-induced double-strand breaks can generate complex structural variants—including large deletions, inversions, and chromothripsis-like events—that may be undetectable with standard short-read sequencing. Such hidden alterations introduce uncertainties in genomic integrity, posing challenges for establishing regulatory-compliant release criteria in clinical-grade T cell products. These unintended events can compromise cell viability and raise long-term safety concerns, especially in the context of large-scale manufacturing. Within this spectrum of solutions, base-editing is best viewed not as a universal requirement but as a complementary option that may be particularly valuable in settings where tighter genomic control or reduced double-strand break–associated alterations are desired. For example, cytosine and adenine base editors can inactivate endogenous TCR genes or modify constant regions to prevent mispairing, while minimizing genotoxic stress (35). Potential module interaction and required QC. We acknowledge that eliminating endogenous TCR chains (TRAC/TRBC disruption) can, in some settings, alter CD3 complex availability and surface assembly dynamics, which may affect the expression level, stability, or functional avidity of the introduced transgenic TCR. Therefore, the editing module should be coupled to a dedicated release/QC check that confirms stable surface transgenic TCR and CD3 expression (such as flow cytometry and pHLA-multimer binding) across manufacturing/expansion, and flags products showing reduced or unstable expression for redesign of editing order-of-operations. Where feasible, orthotopic replacement/targeted insertion at the TRAC locus can further stabilize receptor expression under endogenous regulatory control (36, 37). Because base editors operate without creating double-strand breaks, they minimize p53-mediated stress responses and avoid repair-pathway heterogeneity, resulting in edited T cell populations with far narrower indel distributions and improved lot-to-lot consistency during manufacturing.

In addition to eliminating mispairing, base-editing can be coupled with the installation of “safety switches” such as inducible suicide genes, which allow clinicians to terminate therapy in the event of severe toxicity (38). This layered engineering approach transforms TCR-T products into controllable platforms rather than permanent genetic interventions. Furthermore, the modularity of base-editing ensures compatibility with emerging TCR formats, including STAR, HIT and TRuC, providing flexibility as receptor engineering diversifies (39, 40).

Taken together, we propose that base-editing should be prioritized as a preferred mispairing-mitigation strategy in settings where tighter genomic control and reduced double-strand-break-associated alterations are required, representing a deliberate safety-by-design decision rather than a purely technical optimization. By replacing blunt genome disruption with precise, reversible editing strategies, the field can significantly reduce clinical risk, build regulatory confidence, and accelerate the path toward routine, large-scale application of engineered T cells in oncology.

## 4 Prioritizing public neoantigens

Notably, large-scale expression atlases commonly treat ~0.5 TPM as a practical boundary between background and reproducible baseline expression: for example, Expression Atlas–derived binning schemes classify <0.5 TPM as “below cutoff/absent” and ≥0.5 TPM as “present” (with 0.5–10 TPM labeled as low expression). Consistent with this convention, the Mouse Gene Expression Database (GXD) maps quantile-normalized TPM values to Expression Atlas bins and explicitly uses <0.5 TPM to call genes absent (41). The selection of suitable target antigens remains a central determinant of both efficacy and safety in TCR-T therapy. While personalized neoantigen discovery has generated excitement for its theoretical precision, it is hindered by several practical limitations. Patient-specific pipelines require extensive sequencing, bioinformatic prediction, and individualized manufacturing, resulting in long lead times and high costs (42, 43). Moreover, the heterogeneous nature of tumors means that some private neoantigens may be expressed at low frequency or in subclones, limiting therapeutic durability (44, 45). In addition, the accuracy of neoantigen prediction pipelines remains highly variable, with peptide–MHC binding affinity explaining only a fraction of true immunogenicity. Many predicted epitopes fail to be naturally processed or presented, and even validated epitopes may be lost through immune editing, creating a moving target for personalized therapy. These intrinsic uncertainties limit the reliability and reproducibility of fully individualized neoantigen strategies. These barriers restrict personalized approaches largely to proof-of-concept settings rather than scalable clinical practice.

In contrast, public neoantigens (recurrent hotspot mutations or aberrantly expressed developmental antigens shared across patients) enable the development of standardized TCR-T products when selected using rational prioritization criteria such as population-level HLA coverage, mutation recurrence frequency, predicted antigen processing likelihood, and minimal normal-tissue expression. Examples include recurrent oncogenic mutations such as KRAS G12D/V, TP53 hotspot variants (46), and aberrant expression of developmental antigens like MAGE-A4 or NY-ESO-1 (47). Importantly, several of these targets have already entered clinical trials, with afamitresgene autoleucel (afami-cel), a MAGE-A4–directed TCR-T therapy, achieving regulatory recognition and paving the way for a class of standardized products (48). Such successes highlight the translational advantages of focusing on well-validated, recurrent antigens.

Prioritizing public neoantigens does not eliminate risk, the possibility of low-level expression in normal tissues necessitates rigorous specificity screening. However, when combined with AI-guided prediction and base-editing safeguards, public antigens provide a strong foundation for reproducible and regulatorily favorable products. From a health-system perspective, therapies targeting shared antigens can be manufactured at scale, reducing production variability and improving accessibility. Furthermore, because these targets recur across diverse HLA backgrounds, they create opportunities for pan-population strategies rather than fragmented, patient-specific interventions.

Looking ahead, a rational prioritization framework should balance public antigen development for scalability with personalized discovery for niche indications, ensuring that safety and feasibility are not compromised by overemphasis on individualized pipelines. By aligning therapeutic innovation with population-level practicality, TCR-T therapies can transition from experimental boutique treatments to broadly available modalities that benefit larger patient cohorts. Importantly, this integrated safety pathway is modality-agnostic: the same AI-driven specificity triage and systematic off-target interrogation should be applied to other pHLA-targeting agents, including TCR-mimic antibodies and related bispecific formats, which share analogous cross-reactivity risks (49).

## 5 Integrating AI, genome editing, and neoantigen strategy across TCR-based therapeutic modalities

Individually, these three pillars each address a key safety gap in TCR-T development. However, their true potential lies in a coordinated framework that integrates these elements into a coherent pipeline. Such integration enables the construction of a unified safety architecture in which AI-derived specificity scores inform receptor selection, base-editing ensures genomic fidelity and mispairing control, and public neoantigen prioritization defines antigenic boundaries—together establishing explicit, traceable decision gates that systematically reduce uncertainty throughout the TCR-T development pipeline. The value of such an integrated framework is underscored by the success of engineered T-cell therapies in hematologic malignancies, where well-defined antigens enable consistent clinical benefit (3). Extending similar reliability to solid tumors will require precisely the kind of multi-layered safety engineering proposed here.

A practical roadmap can be envisioned as a three-stage safety pathway. As summarized in **Figure 1**, a practical roadmap is to link these pillars into a staged safety pathway that moves from AI-based triage, through genomically refined effector cells, to shared neoantigen targets, thereby creating explicit decision gates across the TCR-T development pipeline.

Crucially, integration across these stages enhances the credibility of TCR-T programs in the eyes of regulators, payers, and patients. A therapy that demonstrates computationally validated specificity, genetically reinforced safety, and population-level applicability will be viewed not only as innovative but also as trustworthy. Such an approach could accelerate clinical trial approval, streamline manufacturing pipelines, and foster broader adoption within oncology practice. By embedding these complementary strategies into a unified design philosophy, the TCR-T field can move beyond incremental advances. The outcome is a robust translational framework that elevates safety from an afterthought to the central organizing principle of therapeutic innovation.

## 6 Toward safe and scalable TCR-T therapies

The future success of TCR-T therapy will depend on whether the field can reconcile its remarkable precision into predictable, auditable, and regulatorily acceptable safety performance. Although advances in this integrated safety framework mark important progress, they will only achieve clinical impact if embedded within a cohesive, quantitatively defined validation framework. As the clinical history of off-target toxicities has shown, safety cannot rely on isolated innovations; it requires a multi-layered pipeline that rigorously interrogates antigen specificity, genomic integrity, and target selection at every stage of development.

A critical next step is the creation of standardized, multi-modal safety benchmarks that integrate computational predictions with high-throughput functional assays and structural validation. Such benchmarks should not only assess peptide–MHC recognition breadth and cross-reactivity risk, but also quantify editing-associated genomic alterations, establish acceptable thresholds for indel heterogeneity, and define minimal criteria for normal-tissue expression of candidate antigens. Embedding these metrics into manufacturing and release decisions would begin to align TCR-T development with the quality-control paradigms already expected for other advanced therapies.

Regulatory acceptance will hinge on transparent demonstration that engineered receptors are screened against the breadth of the human proteome, edited to eliminate mispairing, and directed toward well-validated targets. At the same time, collaborative efforts between academic groups, biotech companies, and clinical centers will be necessary to expand high-quality datasets, improve predictive models, and accelerate the translation of editing technologies into GMP-compliant platforms.

Most importantly, adopting a safety-first ethos does not diminish therapeutic ambition; rather, it provides the foundation for scalability and accessibility. By anchoring innovation to reliable safeguards, TCR-T therapies can move beyond boutique, patient-specific solutions and evolve into standardized, population-level treatments. Such a transformation would not only improve patient outcomes but also build the regulatory and societal trust required for widespread clinical adoption.
